# Myeloprotection with activated carbon in doxorubicin-treated rats

**DOI:** 10.1016/j.heliyon.2023.e18414

**Published:** 2023-07-18

**Authors:** Bogdan I. Gerashchenko, Veronika V. Sarnatskaya, Kvitoslava I. Bardakhivska, Oleksii S. Sydorenko, Denis L. Kolesnik, Dmytro O. Klymchuk

**Affiliations:** a R.E. Kavetsky Institute of Experimental Pathology, Oncology and Radiobiology (IEPOR), National Academy of Sciences of Ukraine, Vasylkivska Str. 45, Kyiv, 03022, Ukraine; b M.G. Kholodny Institute of Botany, National Academy of Sciences of Ukraine, Tereshchenkivska Str. 2, Kyiv, 01601, Ukraine

**Keywords:** Doxorubicin, Activated carbon, Bone marrow, Hematopoiesis, Myeloprotection, Total nucleated cells, Polychromatic erythrocytes, Acridine orange, Fluorescence intensity, Flow cytometry

## Abstract

Chemotherapy can often cause a variety of side effects including bone marrow (BM) suppression, termed as myelosuppression. Accordingly, facile and effective management of chemotherapy-induced myelosuppression is currently a pivotal task for experimental pathologists and oncologists. Here, we chose to use activated carbon (AC) with an extensive surface area for studying its possible protective effectiveness with respect to BM in doxorubicin (DOX)-treated rats. Spherical AC with an extended surface area up to 4490 m^2^/g was prepared for *per os* (p/o) delivery, whereas for intraperitoneal (i/p) delivery we used the powdered form of AC that was derived from the aforementioned spherical AC. During the monthly treatment of animals with AC and DOX these two components were delivered alternately (not in the same day). After treatment, BM cells were isolated from femurs of sacrificed animals, stained with acridine orange (AO) and analyzed by flow cytometry. Regardless of the route of AC delivery (p/o or i/p), apparent myeloprotection with a possible regenerative effect was observed in animals that received DOX, as evidenced by recovery of the populations of total nucleated cells (TNC) and polychromatic (immature) erythrocytes accompanied by a considerable reduction of the number of apoptotic/dead cells among TNC (≤2.0%). Moreover, as a result of AC administrations, there was a significant increase of AO green and far-red fluorescence intensities in the population of TNC, which is suggestive of the ongoing quantitative and conformational changes in DNA and RNA associated with cell recovery and proliferation. Thus, AC preparations under the present experimental conditions can effectively tackle DOX-induced myelosuppression via mechanisms not necessarily associated with adsorptive detoxification.

## 1 Introduction

Bone marrow (BM) is a sensitive target for a number of anticancer chemotherapeutic drugs that are currently used in oncology practice. Although chemotherapy-induced BM suppression (myelosuppression) is a frequent complication, it does seem obvious that success of chemotherapy may also depend on extent of myelosuppression, which is critical for normal and undisrupted hematopoiesis. Therefore, development of the strategy to counter this problem with the use of effective and relatively inexpensive therapeutic means is of great importance. Activated carbon (AC), which is effective in treatment of various exogenous and endogenous intoxications [1], has also attracted attention of scientists in terms of its capability to promote regenerative processes in some organs and tissues including BM [2]. Using the oral route of intragastric administration in animal models, spherical AC made from the product of pyrolysis of the nitrogen-containing synthetic polymer resin has been studied in terms of its possible use for tackling chemotherapy-induced myelosuppression, and results were promising, showing apparent improvements in BM and peripheral blood cell counts [[3], [4], [5]]. Similar to nitrogen-containing AC, AC representing pure carbon made from the product of pyrolysis of the porous phenol-formaldehyde resin has also been shown to improve hematopoiesis affected by a chemotherapy drug, according to the peripheral blood cells counting data [6]. This myeloprotective property of AC was observed with respect to such chemotherapy drugs, as cyclophosphamide [3], methotrexate [3], and melphalan [[4], [5], [6]]. Both, cyclophosphamide and melphalan are alkylating agents causing DNA crosslinks, while methotrexate is an antimetabolite that antagonizes the actions of folate (vitamin B_9_) required for DNA and RNA synthesis, and cell division. In the current study, we chose to use another chemotherapy drug – doxorubicin (DOX), that belongs to the class of anthracycline antibiotics and that is also characterized by a significant myelosuppressive effect. As regards the mechanisms of anticancer effect of DOX, besides the inhibition of DNA synthesis via intercalation into DNA, it interferes in topoisomerase II activity and contributes to generation of free radicals in amounts capable of damaging cells [7]. Here, nitrogen-containing AC with an extremely large surface area (larger than in carbons in aforementioned studies) was prepared and used in either *per os* (p/o) or intraperitoneal (i/p) delivery to assess its myeloprotective properties in DOX-treated rats.

Although the cytotoxic insult to BM can indirectly be assessed by peripheral blood cell counts, BM examinations provide the direct evidence of what is exactly occurring in this vitally important hematopoietic tissue. For this purpose, the use of rapid and reliable techniques is of great importance. Accordingly, in this study, flow cytometry (FCM) of BM cells stained with acridine orange (AO) was chosen to assess myeloprotection by AC preparations in DOX-treated rats. AO is a metachromatic dye that interacts with DNA and RNA producing the dual emission spectra with peaks at 530 and 640 nm, respectively, if it is excited at 488 nm [8]. Based on these fluorescence characteristics and the differences in AO uptake by BM cells, the FCM approach has been developed to discriminate BM cell populations in laboratory animals either intact or pharmacologically treated [[9], [10], [11]]. FCM analysis data obtained in this study were further supplemented with the data on cell stiffness-related deformability using laser scanning confocal microscopy (LSCM).

## 2 Materials and methods

### 2.1 AC preparations and morpho-dimensional characterization methods

Depending on the route of AC administration (p/o or i/p), two forms of AC were used. The first form of AC, which is designed for p/o administration, represented beads of diameters 150–250 μm made from the product of pyrolysis of nitrogen-containing synthetic (4-vinylpyridine-styrene-divinylbenzene copolymer) resin [12]. Before use, AC beads underwent an additional activation procedure – steam activation in fluidized bed at 800–950 °C within 1–2 h, resulting in 1.5–2.0-fold increase of the surface area (up to 4490 m^2^/g) with concomitant decrease of the bulk density (up to 0.095 g/cm^3^). Surface area was determined by measuring adsorption of nitrogen at −196 °C with Brunauer–Emmett–Teller method using gas adsorption analyzer Autosorb 6 (Quantachrome Instruments, Boynton Beach, FL). To obtain the second form of AC to be used for i/p administration, the first form of AC (beads), that was additionally activated under conditions mentioned above, underwent mechanical grinding, resulting in the fine powder of the bulk density of 0.037 g/cm^3^. The technique of obtaining fine powders from the beads of AC has been previously described [13]. In brief, these beads were crushed with an automatic multichannel milling system “MKM-300H” (“МИЛЛКОМ” Ltd, Kharkiv, Ukraine) at 60-Hz angular frequency of separator for 20 min.

Both forms of AC, beads and powder, were coated with a thin layer of gold (7–13 nm) at 1.5 kV and 10 mA for 2 min using a fine coat ion sputter JFC-1100 (JEOL Ltd, Tokyo, Japan) followed by scanning electron microscopy (SEM) with a JSM-6060LA system (JEOL Ltd, Tokyo, Japan). The images were collected at the accelerating voltage of 30 kV.

The particles of AC powder were suspended in pure water (200 μg/ml) and their size distribution was determined in 1-ml aliquots by means of dynamic light scattering (DLS) using a Malvern Instruments ZetaSizer (Worcestershire, UK) equipped with He–Ne laser (25 mW, λ = 633 nm) with an angle detection of 90° at 22 °C.

### 2.2 Experimental design

Adult female Wistar rats (200–220 g) were used in the experiment approved by the Committee of Bioethics at IEPOR under the ethical approval № 1a of February 16, 2017 and performed according to the rules and requirements of the European Convention for the Protection of Vertebrate Animals used for Experimental and Other Scientific Purposes. Before the experiment, the animals were randomly assigned to four groups. In the group 1 (n = 5), DOX under the brand name Doxorubicin Ebewe (EBEWE Pharma Ges.m.b.H. Nfg.KG, Austria) was delivered i/p twice a week with single doses of 3.25 mg/kg (cumulative dose: 26 mg/kg). In the group 2 (n = 4), on the next day after DOX administrations, the particles of AC powder suspended in isotonic saline were delivered i/p twice a week with single doses of 12.5 mg/kg (cumulative dose: 100 mg/kg). As regards p/o delivery of AC in the group 3 (n = 4), 2 cm^3^ of AC beads were mixed with about 5.5 cm^3^ of freshly cooked oatmeal and given to rats in the morning under fasting conditions next two days after DOX administrations, so the daily dose of AC was 1 g per 1 kg of rat's body weight. Two hours later, oatmeal with AC beads was replaced with a regular food provided by vivarium. The group 4 (n = 5) represented intact animals, who received placebo, i.e. isotonic saline with no DOX. On day 3 after the last administration of AC preparations BM cells were isolated from femurs of sacrificed animals as described [9].

### 2.3 Specimen fixation and staining

Before FCM analysis, BM samples that were collected and stored at + 4–6 °C underwent fixation and staining procedures [9]. These samples were resuspended by vortexing and centrifuged at 300 g for 5 min followed by washing cells in 5 ml of phosphate buffered saline (PBS; pH 7.2–7,4) using centrifugation at 300*g* for 5 min. Supernatants were discarded followed by resuspension of cells in 2 ml of PBS. Cell aggregates were dissociated by gentle syringing of the suspension through a 21-gauge needle. While vigorous vortexing, 0.2 ml of cell suspension was added to 5 ml of fixative solution 1% glutaraldehyde (v/v) in PBS with 30 μg/ml of sodium dodecyl sulfate. Cells were fixed for 5 min and then centrifuged at 300 g for 5 min. After resuspension in 0.5 ml of PBS, 0.2 ml of cell suspension was added to 0.4 ml of ice-cold solution A followed by adding of 1.2 ml of ice-cold solution B that were prepared as follows. Solution A was prepared by dissolving in 100 ml H_2_O (final volume) of 0.1 ml Triton X-100, 8 ml 1.0 N HCl, and 0,877 g NaCl, while solution B represented the mixture of 37 ml 0.1 M citric acid and 63 ml 0.2 M Na_2_HPO_4_ (pH 6.0) with added 0.877 g NaCl, 34 mg EDTA disodium salt, and 0.6 ml of stock solution (1 mg/ml) of AO (Sigma-Aldrich, St. Louis, MO). While shaking, cells were stained on ice in the dark for 30 min. Cells were then centrifuged at 300 g for 5 min to remove supernatants and resuspend them in 1 ml of PBS with gentle syringing through a 21-gauge needle.

### 2.4 FCM analysis

Samples were analyzed with a DxFLEX flow cytometer (Beckman Coulter Life Sciences, Indianapolis, IN) using 50-mW blue laser (488 nm). In general, FCM analysis was performed with parameter settings similar to those as proposed for AO-stained BM cells [9]. Cell size-related forward light scatter (FSC) and intracellular granularity-related side light scatter (SSC) signals were collected in a linear mode. Fluorescence signals of AO presumably bound to DNA and RNA were collected in a logarithmic mode through 525/40 and 690/50 band-pass filters, respectively. The acquisition rate was not higher 1000 cells per second at a constant flow rate of 30 μl/min. At least 2.5 × 10^4^ events were collected for each sample. Analysis of the data was performed using CytExpert for DxFLEX software (Beckman Coulter, version 2.0). Cells were gated on FSC-Height vs. SSC-Height histograms to eliminate debris and aggregates from analysis, although their numbers were very low according to microscopic observations. According to this FCM protocol, the total nucleated cells (TNC), polychromatic (PCE) and normochromatic erythrocytes (NCE) can be discriminated. In the population of TNC, myeloid cells can be discriminated from erythroid nucleated cells (ENC) based on the difference of their sizes, as proposed [14].

### 2.5 LSCM analysis

The rest of AO-stained BM cells were used for LSCM with the aim to assess deformability of nucleated cells that relates to their stiffness. Before LSCM, 15-μl aliquots of cell suspension from one of the samples of each group were placed on the surface of microscope slides followed by careful covering them with 18 × 18 mm coverslips to ensure uniform spreading of cells with no air bubbles. A Zeiss LSM 5 PASCAL confocal microscope (Carl Zeiss, Jena, Germany) equipped with a Plan-NEOFLUAR 40x/0.75 dry objective was used. Argon laser (488 nm, 30 mW) was selected for excitation of AO. Green fluorescence was detected through a 505–530 nm band-pass filter, whereas red fluorescence was detected through a 560 nm long-pass filter. The images of randomly selected nucleated cells were acquired and analyzed with a Laser Scanning Microscope LSM 5 PASCAL software (Carl Zeiss, version 3.2). These images consisted of a confocal series (z-series) of sequential scans with 0.5-μm step size directed from top to bottom of cells. Scan speed and zoom were set on seven and two, respectively. To ensure the acquisition of high quality images, the pinhole size for both channels was set on 125 μm, providing a superior spatial resolution and synchronous collection of green and red fluorescence signals through the entire stack of optical sections. Amplifier and gain settings were adjusted such that green and red fluorescence intensities were nearly equal, ensuring equal acquisition of z-series in both channels. All these and other settings, including intensity of the laser, were kept unchanged at the time of image acquisition.

### 2.6 Statistical analysis

Significance of the differences between cohorts of the data was assessed by Student's *t*-test and by one-way ANOVA test using an OriginPro 7.5 software (OriginLab Corporation, Northampton, MA). Probability (*P*) values of less than 0.05 were considered as statistically significant.

## 3 Results

### 3.1 Morpho-dimensional characteristics of AC preparations

**Fig. 1:**
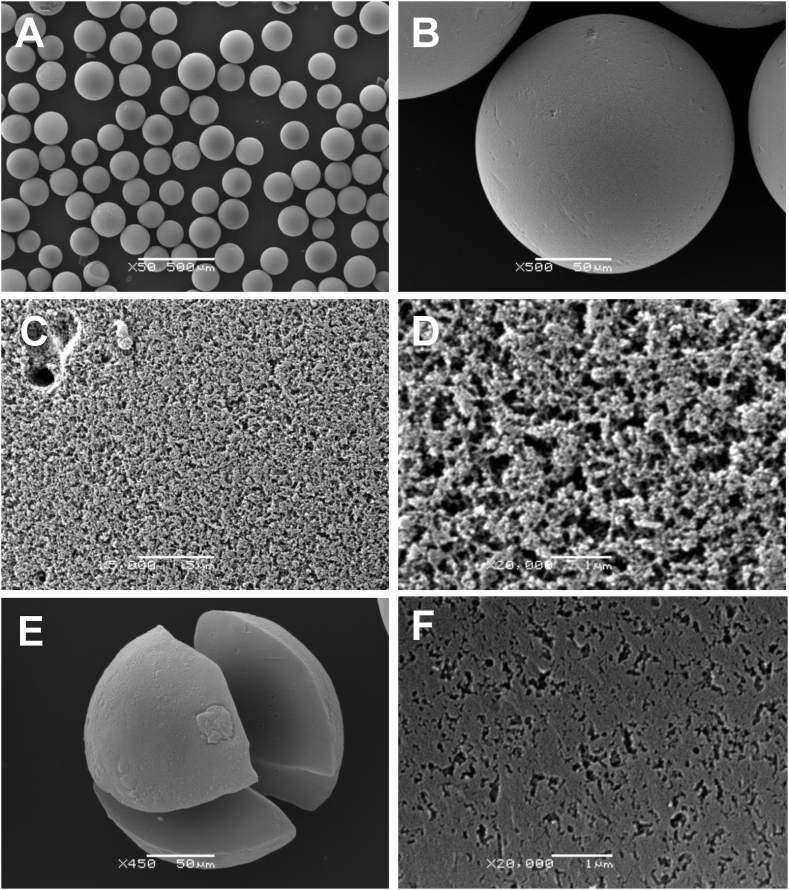
SEM images of AC beads demonstrating their outer (**A**–**D**) and inner (**E**–**F**) morphology at different magnifications. Scale bars: 500 μm (in **A**), 50 μm (in **B**), 5 μm (in **C**), 1 μm (in **D**), 50 μm (in **E**), and 1 μm (in **F**).

Fig. 1 presents the outer and inner morphology of AC beads at different magnifications. All of them are spherically shaped having diameters of 150–250 μm (Fig. 1A) with seemingly smooth and solid surface (Fig. 1B). The surface of AC beads consists of a great number of open pores (Fig. 1C and D), resembling those in carbon adsorbents, termed as HSGD (HemoSorbent Granulated Deliganding [15]), which is produced from the same type of nitrogen-containing synthetic resin. Examination of the inner composition of AC beads revealed well developed, dense and uniform porosity (Fig. 1E and F), that similarly to HSGD may have predominant dimensions of mesoporous range [16]. In fact, these AC beads, in addition to very large specific surface area (4490 m^2^/g), were found to be characterized by an exceptionally high total pore volume of 5.29 cm^3^/g with an average pore diameter of 4.71 nm, which is extremely rare for activated and other porous carbons described elsewhere to date [17,18].

**Fig. 2:**
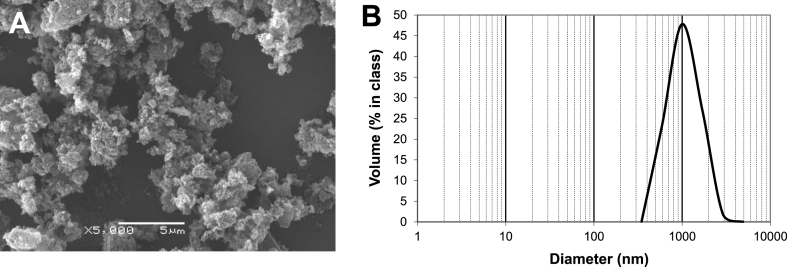
SEM image of the fine powder particles of AC (**A**) and their size distribution based on DLS data (**B**). Scale bar: 5 μm.

Fig. 2A presents SEM image of the particles obtained by mechanical grinding of AC beads. These variously sized and irregularly shaped particles stick together likely due to electrostatic precipitation, forming friable flakes. In the aqueous suspension, hydrodynamic diameters of AC particles ranged from 0.35 to 4.9 μm with the mean value of 1.13 μm (Fig. 2B).

### 3.2 FCM profiles of AO-stained BM samples of different groups of animals

**Fig. 3:**
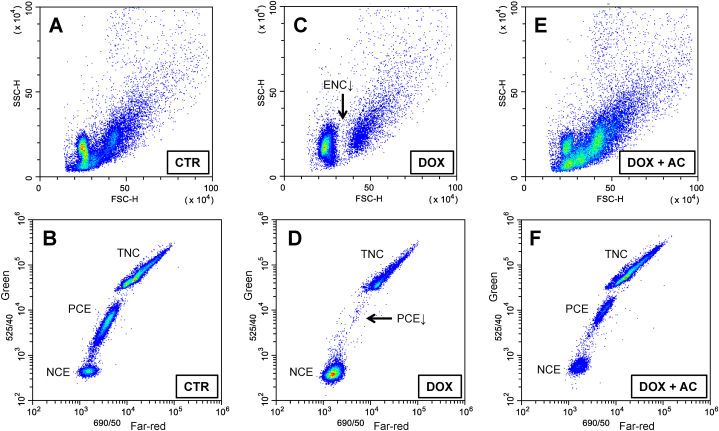
FCM profiles of three representative AO-stained BM samples: CTR – intact control (**A** and **B**), DOX – rats treated with doxorubicin (**C** and **D**), DOX + AC – rats treated with doxorubicin and activated carbon that was delivered via i/p route (**E** and **F**). The top three plots (**A**, **C**, and **E**) represent FSC-Height vs. SSC-Height signal distribution, while the corresponding bottom three plots (**B**, **D**, and **F**) represent green vs. far-red AO fluorescence intensity signal distribution distinctly discriminating total nucleated cells (TNC), polychromatic erythrocytes (PCE) and normochromatic erythrocytes (NCE). The arrows show depletion of PCE and erythroid nucleated cells (ENC) in BM due to DOX-treatment. (For interpretation of the references to colour in this figure legend, the reader is referred to the Web version of this article.)

**Fig. 4:**
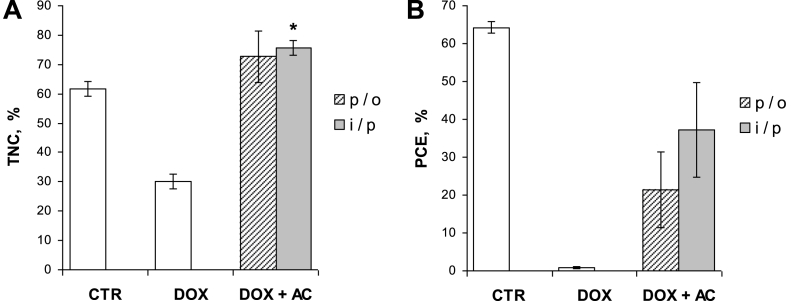
Comparison of TNC (**A**) and PCE (**B**) populations between AO-stained BM samples of different groups of animals based on FCM analysis. The difference of TNC population in DOX + AC group with p/o route of AC delivery, compared with that in CTR group, was insignificant (*P* > 0.05; panel **A**), whereas the difference of TNC population in DOX + AC group with i/p route of AC delivery, compared with that in CTR group, was significant (*P* < 0.05; shown by asterisk in panel **A**). Data presented as the mean ± standard error of the mean.

**Fig. 5:**
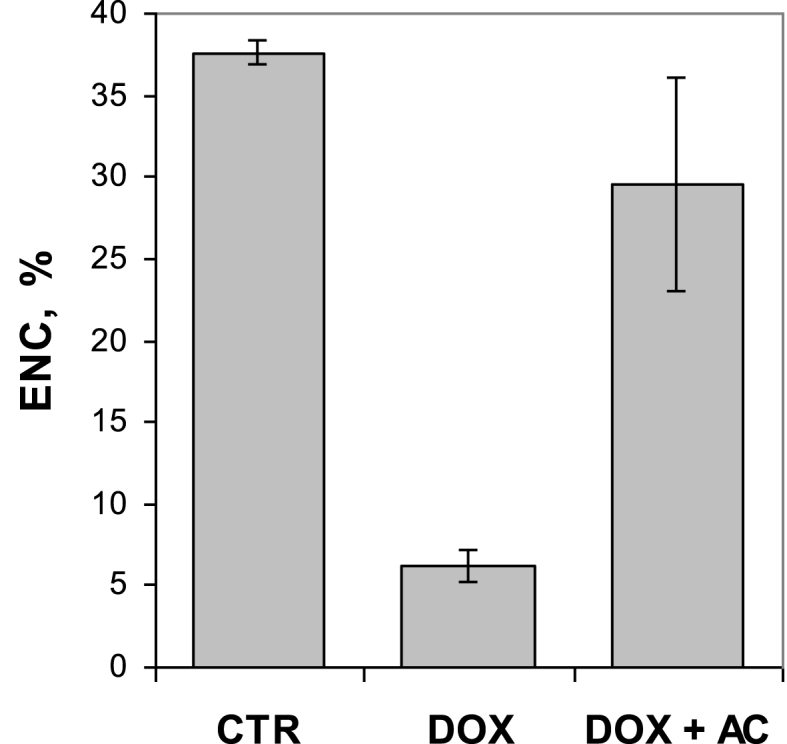
Comparison of erythroid nucleated cell (ENC) populations within TNC in AO-stained BM samples of different groups of animals based on FCM analysis. In the group DOX + AC, AC was delivered via i/p route. The difference of ENC population in DOX + AC group, compared with that in CTR group, was insignificant (*P* > 0.05). Despite the route of AC administration, there was not any significant difference in the data (not shown). Data presented as the mean ± standard error of the mean.

As expected, the monthly course of DOX administration led to a significant and persistent myelosuppression based on the analysis of BM cells by FCM. This is evidenced by a substantial decrease of the population of TNC having larger FCS and fluorescence (far-red and green) signals accompanied by depletion of populations PCE and ENC (Fig. 3). Interestingly, regardless of the route of AC administration (either i/p or p/o) in rats treated with DOX, there was restoration of light scatter and fluorescence signal distribution patterns resembling those of the intact control (Fig. 3). The results of quantitative analysis of TNC and PCE populations are presented in Fig. 4A and B, respectively. As shown in Fig. 4A, AC administrations in DOX-treated animals resulted in a significant increase of TNC population: 75.6 ± 2.5% (i/p route) and 72.6 ± 8.8% (p/o route), compared with TNC population of DOX-treated animals that did not receive AC (30.1 ± 2.6%). Notably, the population of TNC in DOX + AC groups was even 1.2-fold larger than that of the intact control (*P* < 0.05 if AC delivered i/p, whereas *P* > 0.05 if AC delivered p/o). Moreover, in both DOX + AC groups the apoptotic/dead cells accounted for ≤2% of TNC, whereas their number in DOX group was considerably higher (32–64%), as these cells are morphologically different and less bright, especially in green fluorescence, than the rest of TNC (not shown). As for PCE, the apportionment of these cells was calculated within the whole erythrocyte population of BM that includes NCE as well (Fig. 3). As shown in Fig. 4B, AC administrations in DOX-treated animals also resulted in a significant increase of PCE population: 37.3 ± 12.5% (i/p route) and 21.3 ± 10.1% (p/o route), compared with PCE population of DOX-treated animals that did not receive AC (0.9 ± 0.3%). However, these values were not as high as in CTR group that showed 63.7 ± 2.2%. While analysing within TNC population the apportionments of ENC, that are typically smaller than other types of nucleated cells residing in BM [11,19], these apportionments were found to concordantly change with those of PCE (Fig. 5 vs. Fig. 4B).

**Fig. 6:**
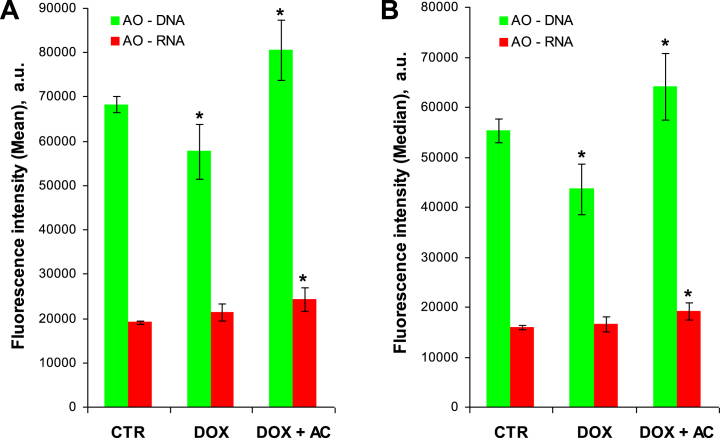
The mean (**A**) and the median (**B**) of green and far-red fluorescence intensity signal distributions for TNC populations (excluding apoptotic/dead cells) of AO-stained BM samples. In the group DOX + AC, AC was delivered via i/p route. Significant differences (*P* < 0.05) in the data while comparing treatment groups with CTR are denoted with asterisks. Data presented as the mean ± standard deviation. (For interpretation of the references to colour in this figure legend, the reader is referred to the Web version of this article.)

Of further interest, AO fluorescence intensity signal distributions were analyzed for TNC populations, excluding apoptotic/dead cells. The conjunctive mean and median values of these distributions were found to be well correlated that are presented in Fig. 6A and B, respectively. In comparison with CTR group, there was 1.2–1.3-fold decrease of green fluorescence in DOX group. However, in DOX + AC group the green fluorescence was 1.4–1.5-fold and 1.2-fold brighter than that in DOX and CTR groups, respectively. Contrary to the green fluorescence, there was no decrease of far-red fluorescence in DOX group. However, similarly to the green fluorescence, the far-red fluorescence in DOX + AC group was 1.2–1.3-fold brighter than that in CTR group.

### 3.3 Cell stiffness-related deformability data

**Fig. 7:**
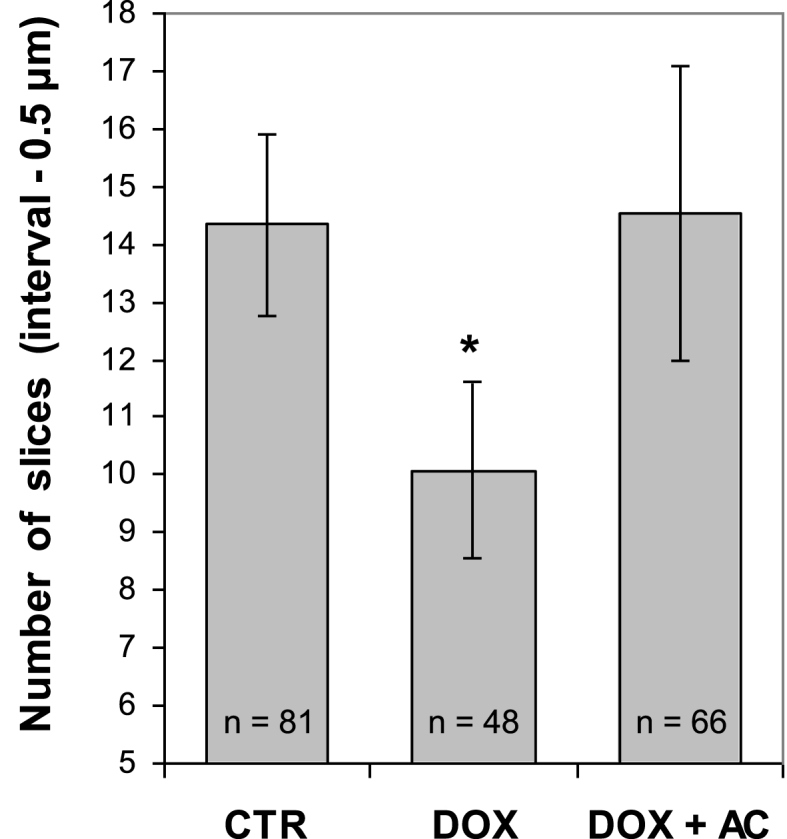
Assessment of cell stiffness-related deformability by comparison of the average numbers of optical slices in z-series of nucleated cells in three representative AO-stained BM samples: 1) CTR – intact control, 2) DOX – rats treated with doxorubicin, 3) DOX + AC – rats treated with doxorubicin and activated carbon (AC was delivered via i/p route). There is a significant difference (*P* < 0.05; shown by asterisk) while comparing the data in DOX group with the data in CTR and DOX + AC groups. Data presented as the mean ± standard deviation.

As shown in Fig. 7, deformability of BM nucleated cells that directly relates to their stiffness can be assessed by quantification of the numbers of sequential scans (optical slices) composing LSCM z-series. Notably, cellular deformability in BM of rat that received DOX + AC was similar to that in BM of intact rat (CTR), as confirmed by the equality of the average numbers of optical slices (within 14–15) in these two samples, whereas cellular deformability in BM of rat that received DOX without AC was 1.4-fold lower than in samples CTR and DOX + AC (*P* < 0.05). Despite the route of AC administration, there was not any significant difference in the data (not shown).

## 4 Discussion

Thus, FCM analysis data showed that AC used in this study, regardless of the route of administration, is capable of protecting hematopoietic BM cells, as evidenced by recovery of TNC and PCE populations accompanied by a considerable reduction of the number of apoptotic/dead cells among TNC. This finding is indicative of the expansion of TNC with accelerated maturation of erythrocytes, despite a distinct susceptibility of erythropoiesis to DOX treatment, as evidenced by a drastic reduction of PCE (Fig. 3, Fig. 4B) and ENC populations (Fig. 5). Suzuki et al. [20] suggested that redistribution of erythrocytes towards NCE, causing reduction in PCE/NCE ratio, is most likely due to mutagen-induced rapid differentiation and enucleation of erythroblasts resulting in accumulation of NCE in BM instead of entering the peripheral blood stream. On the other hand, Von Lebedur and Schmid [21] reported that the aforementioned redistribution of erythrocytes can be explained by a partial depletion of BM cavities of nucleated blood cell precursors with subsequent retention of newly formed NCE and inundation with peripheral blood. Erythropoietic susceptibility has also been shown with respect to DNA-damaging agent of a physical origin, such as ionizing radiation (IR), [22,23]. While assessing the apportionments of PCE population depending on the route of AC delivery, we revealed that at i/p delivery this population was 1.75-fold larger than that at p/o delivery, although this difference was insignificant (*P* > 0.05, Fig. 4B). Perhaps, i/p-delivered AC can more readily protect hematopoiesis. It is worth noting that both, DOX and AC, were delivered via i/p route, but not in the same day, thus avoiding the interference with one another in view of the fact that AC is a strong adsorbent. In this situation, one can assume that during the time between DOX administrations, AC, being in the peritoneal milieu, becomes much less adsorptive or not adsorptive at all due to saturation of its surfaces with a variety of biological substances. On the other hand, AC by diminishing of certain types of low weight biomolecules (<5 kDa) theoretically may favour hematopoiesis mainly at the expense of erythropoiesis, based on the results of *in vitro* study by Murate et al. [24], showing an excellent erythroid colony-supporting ability of fetal bovine serum treated with AC. Contrary to i/p-delivered AC, p/o-delivered AC is likely to diminish such molecules to a lesser extent, because the gastrointestinal tract milieu also contains food nutrients and its metabolites that can be adsorbed by AC, thus saturating its surfaces and resulting in less pronounced effect on recovery of PCE population.

Nevertheless, in spite of the differences in AC delivery, effects were in general similar, suggesting the involvement of similar mechanisms, even though the different forms of AC were used (spheres or powder). The fact that TNC population in DOX + AC groups was 1.2-fold larger than that in CTR group (Fig. 4A) is likely due to ongoing regenerative processes characterized by an active cell proliferation. Moreover, this gain of TNC population is concomitant with the gain of the magnitude of fluorescence intensity signals (green and far-red) from TNC of corresponding groups (Fig. 6), suggesting an active synthesis of nucleic acids (DNA and RNA) and the presence of conformational changes, particularly in chromatin and DNA, associated with promotion of cell recovery and proliferation. The fact that these TNC after AC treatment keep on maintaining the stably viable state can further be supported by the finding that the ratios of green to far-red fluorescence intensity signals were similar to those in CTR, regardless of whether the mean or the median of fluorescence intensity signal distributions was used for calculating the ratios (Table 1). The FCM data that demonstrate a distinct myeloprotective effect of AC (Fig. 4, Fig. 5, Fig. 6) are generally consistent with supplementary LSCM data (Fig. 7), which is indicative of the interrelation between the viability/proliferation state of nucleated cells and their stiffness.

**Table 1:** The ratios of green to far-red fluorescence intensity signals from TNC of AO-stained BM samples of different groups of animals that were calculated using the average values of the mean and the median of fluorescence intensity signal distributions shown in Fig. 6.

	Mean	Median
CTR	3.56	3.49
DOX	2.70	2.63
DOX + AC	3.32	3.35

To date, the issue on how AC is capable of protecting hematopoiesis largely remains unresolved. Hypothetically, in addition to possible diminishing and/or inactivation of key molecules responsible to hematopoietic regulation, AC may interact with peritoneal or intestinal immune cells, initiating signaling pathways to protect BM cells from genotoxic stress and promote hematopoiesis. One can also assume that AC micro-/nanoparticles via interaction with mononuclear phagocyte system (MPS, formerly known as reticuloendothelial system) are capable of modulating the microenvironment of hematopoietic stem cells (HSCs), favouring their prompt recovery, as suggested by Mori and Ito [25], performing carbon black treatments of either whole body irradiated mice or unirradiated mice bearing irradiated BM cells. In those experiments carbon particles (20–50 nm) were delivered intravenously, and some part of them can presumably accumulate in the BM, namely in special capillaries (sinusoids), whose wall is composed of flat endothelial cells, as evidenced by the presence of visible amounts of carbon in sinusoids even at 5 min after injection [26]. The possibility remains that in our study AC particles can also be transported to the BM, if they do cross the intestinal and peritoneal barriers with or without the help of phagocytes. If they do cross, the mechanisms underlying this process could be particle size- and charge-dependent [27]. The overwhelming amount of micro-/nanoparticles may be present on the surface of p/o-delivered AC beads, which is characteristic of many types of uncoated granular AC [28]. It is worthy to note that AC particles can be ingested by sinusoidal macrophages capable of transporting them to BM parenchyma [29,30], although, as reviewed in Ref. [31], AC nanoparticles can also be transported via endothelial structures, such as caveolae, fenestrae and transcellular channels, and this type of particle transportation is bidirectional (to and from the parenchyma). As regards sinusoidal macrophages, they may be required for the maintenance of homeostasis of hematopoietic niches with HSCs that are located in *peri*-sinusoidal areas [32,33]. Those macrophages that survived irradiation of lethal doses and chemotherapy drug treatments are supposed to be involved in restoration of HSC niches [33], and the role of AC particles in modulating this process could be critically important [34].

The next possible action of AC in protecting hematopoiesis in DOX-treated animals is in that it could potentially scavenge DOX-induced generation of reactive oxygen species (ROS), such as hydroxyl radical (^•^OH) and superoxide (O_2_^•−^), owing to the presence of the oxygen-containing groups on AC surface. This assumption is based on the findings that carbon nanomaterials functionalized with the oxygen-containing groups can combat the oxidative stress associated with irradiation and various pathologies [[35], [36], [37], [38], [39], [40]]. In a recent study with rats exposed to CCl_4_ the oxidative stress manifestations were effectively managed by p/o delivery of AC formulations prepared from carbonized phenol-formaldehyde resin [41].

AC-assisted protection of hematopoiesis has been also observed in the studies with IR-exposed laboratory animals in view of the possible mechanisms suggested above. This effect was significant regardless of how the animals were treated with AC: hemoperfusion [42,43] or enterosorption (p/o route) [43,44]. Supposedly, the effective myeloprotection with a subsequent BM recovery may influence survival rates of irradiated animals, according to the finding that a single-course low-volume hemoperfusion with uncoated spherical AC resulted in 60–70% increase in survival of dogs acutely irradiated with X-rays of the dose of 5.25 Gy, whereas survival without hemoperfusion was only 3% [42]. Curiously, even in the study with intact animals, such as intact old rats, the course of enterosorption with AC led to the increase of their mean lifespan (up to 43%) along with demonstration of the tendency to normalization of some metabolic parameters and ultrastructure of various organs [45]. One can only assume that the positive changes may occur in hematopoiesis as well, because no data on this issue were provided in that study.

Finally, it is worthy to note that hematopoiesis may also be affected by a progressively growing cancer not treated with a chemotherapy drug, as confirmed experimentally [46]. In this regard, it seems interesting to explore AC with or without chemotherapy in terms of its possible effects on hematopoiesis and cancer as well in tumor-host associations.

## 5 Conclusions

Despite the route of administration (i/p or p/o), AC preparations used in this study can effectively tackle DOX-induced myelosuppression via mechanisms not necessarily associated with an adsorptive detoxification. AC treatments sustain the viability and regeneration of BM cells characterized by an active cell proliferation, as evidenced by concomitant increases of the population of TNC and TNC's AO fluorescence intensity signals (green and far-red). Erythropoiesis, most susceptible to treatment with DOX, is remarkably protected and recovered by AC treatments, especially if AC is delivered via i/p route. FCM is a precise and informative approach for studying myeloprotective effects of AC. The results of this study can be of interest for researchers working on development of facile and effective means to manage chemotherapy side effects.

## Author contribution statement

Bogdan Gerashchenko: Performed the experiments; Analyzed and interpreted the data; Wrote the paper.

Veronika V. Sarnatskaya: Kvitoslava I. Bardakhivska: Conceived and designed the experiments; Performed the experiments.

Oleksii S. Sydorenko: Contributed reagents, materials, analysis tools or data.

Denis L. Kolesnik: Performed the experiments; Analyzed and interpreted the data.

Dmytro O. Klymchuk: Analyzed and interpreted the data.

## Data availability statement

Data included in article/supp. material/referenced in article.

## Additional information

No additional information is available for this paper.

## Declaration of competing interest

The authors declare that they have no known competing financial interests or personal relationships that could have appeared to influence the work reported in this paper.
